# The cost of diagnostic uncertainty: a prospective economic analysis of febrile children attending an NHS emergency department

**DOI:** 10.1186/s12916-019-1275-z

**Published:** 2019-03-06

**Authors:** Simon Leigh, Alison Grant, Nicola Murray, Brian Faragher, Henal Desai, Samantha Dolan, Naeema Cabdi, James B. Murray, Yasmin Rejaei, Stephanie Stewart, Karl Edwardson, Jason Dean, Bimal Mehta, Shunmay Yeung, Frans Coenen, Louis W. Niessen, Enitan D. Carrol

**Affiliations:** 1 Institute of Infection and Global Health, University of Liverpool, 8 West Derby St, Liverpool, L69 7BE UK; 2 Infectious Diseases Department, Alder Hey Children’s NHS Foundation Trust, Eaton Road, Liverpool, L12 2AP UK; 3 Alder Hey Children’s NHS Foundation Trust, Eaton Road, Liverpool, L12 2AP UK; 4 The Royal Liverpool University Hospital, Prescot St, Liverpool, L7 8XP UK; 5 Medical Statistics Unit, Department of Clinical Sciences, Liverpool School of Tropical Medicine, Pembroke Place, Liverpool, L3 5QA UK; 6 Royal Derby Hospital, Uttoxeter Road, Derby, DE22 3NE UK; 7 Royal Bolton Hospital, Minerva Road, Farnworth, BL4 0JR UK; 8 School of Medicine, University of Liverpool, Cedar House, Liverpool, L69 3GE UK; 9 Queen Elizabeth Hospital, Mindelsohn Way, Birmingham, B15 2TH UK; 10 Pinderfields District General Hospital, Aberford Road, Wakefield, WF1 4DG UK; 11 Wirral University Teaching Hospital, Arrowe Park Road, Wirral, CH49 5PE UK; 12 Information Department, Alder Hey Children’s NHS Foundation Trust, Eaton Road, Liverpool, L12 2AP UK; 13 Finance Department, Alder Hey Children’s NHS Foundation Trust, Eaton Road, Liverpool, L12 2AP UK; 14 Emergency Department, Alder Hey Children’s NHS Foundation Trust, Eaton Road, Liverpool, L12 2AP UK; 15 Department of Clinical Research, MARCH Centre for Maternal, Adolescent, Reproductive and Child Health, London School of Hygiene and Tropical Medicine, Keppel Street, London, WC1E 7HT UK; 16 Department of Computer Science, University of Liverpool, Ashton Building, Ashton Street, Liverpool, L693BX UK; 17 Department of International Public Health and Clinical Sciences, Liverpool School of Tropical Medicine and University of Liverpool, Liverpool, UK; 18 Department of International Health, Johns Hopkins Bloomberg School of Public Health, Baltimore, MA USA

**Keywords:** Febrile, Fever, Pyrexia, Children, Health economics, Cost of illness, Antibiotics, United Kingdom

## Abstract

**Background:**

Paediatric fever is a common cause of emergency department (ED) attendance. A lack of prompt and definitive diagnostics makes it difficult to distinguish viral from potentially life-threatening bacterial causes, necessitating a cautious approach. This may result in extended periods of observation, additional radiography, and the precautionary use of antibiotics (ABs) prior to evidence of bacterial foci. This study examines resource use, service costs, and health outcomes.

**Methods:**

We studied an all-year prospective, comprehensive, and representative cohort of 6518 febrile children (aged < 16 years), attending Alder Hey Children’s Hospital, an NHS-affiliated paediatric care provider in the North West of England, over a 1-year period. Performing a time-driven and activity-based micro-costing, we estimated the economic impact of managing paediatric febrile illness, with focus on nurse/clinician time, investigations, radiography, and inpatient stay. Using bootstrapped generalised linear modelling (GLM, gamma, log), we identified the patient and healthcare provider characteristics associated with increased resource use, applying retrospective case-note identification to determine rates of potentially avoidable AB prescribing.

**Results:**

Infants aged less than 3 months incurred significantly higher resource use than any other age group, at £1000.28 [95% CI £82.39–£2993.37] per child, (*p* < 0.001), while lesser experienced doctors exhibited 3.2-fold [95% CI 2.0–5.1-fold] higher resource use than consultants (*p* < 0.001). Approximately 32.4% of febrile children received antibiotics, and 7.1% were diagnosed with bacterial infections. Children with viral illnesses for whom antibiotic prescription was potentially avoidable incurred 9.9-fold [95% CI 6.5–13.2-fold] cost increases compared to those not receiving antibiotics, equal to an additional £1352.10 per child, predominantly resulting from a 53.9-h increase in observation and inpatient stay (57.1 vs. 3.2 h). Bootstrapped GLM suggested that infants aged below 3 months and those prompting a respiratory rate ‘red flag’, treatment by lesser experienced doctors, and Manchester Triage System (MTS) yellow or higher were statistically significant predictors of higher resource use in 100% of bootstrap simulations.

**Conclusion:**

The economic impact of diagnostic uncertainty when managing paediatric febrile illness is significant, and the precautionary use of antibiotics is strongly associated with increased costs. The use of ED resources is highest among infants (aged less than 3 months) and those infants managed by lesser experienced doctors, independent of clinical severity. Diagnostic advances which could increase confidence to withhold antibiotics may yield considerable efficiency gains in these groups, where the perceived risks of failing to identify potentially life-threatening bacterial infections are greatest.

## Background

Fever is a common cause of presentation to paediatric emergency departments (EDs) [1], accounting for ~ 20% of all visits [2], but despite its frequent occurrence, the aetiology of fever is diverse [3]. Most children with fever will suffer from self-limiting viral illnesses; however, viral, bacterial, and severe bacterial infections (SBIs) may result in almost identical clinical presentations in infants, making diagnosis based on presentation, history, and clinical judgement alone a difficult task.

While a clear focus of bacterial infection may be present with presentations of acute otitis media (AOM) or urinary tract infection (UTI), occult bacteremia can also occur in children who appear otherwise well, and fever without focus is a common presentation, particularly so in those aged < 36 months [4–6]. However, occurring in as few as 1% of febrile children [4, 5], these ‘hidden’ bacterial infections represent a needle in the haystack, and the challenge for clinicians is to accurately identify children at risk of bacterial infections. While it is possible that they may resolve spontaneously, for those in whom they do not, life-threatening and potentially life-changing complications can develop [4, 7, 8], with adverse outcomes in each survivor of severe meningococcal disease resulting in lifelong treatment costs of ~ £1.3m [9].

As a result, a cautious stepped approach to the management of the febrile child is common, characterised by extended periods of observation, investigations, radiography, and the precautionary use of antibiotics, often prior to definitive evidence of bacterial foci [10]. Unfortunately, such interventions are invasive, can be painful, and are likely to prolong a child’s visit to the ED, contributing to extended ED waiting times and driving the use of scarce ED healthcare resources.

The test currently providing the greatest degree of certainty in diagnosing invasive bacterial infections, the blood culture, typically takes 12–48 h to provide results, has a sensitivity of just 30–40% [11], and a significant false positive rate due to contamination with commensal bacteria from the skin and mucosal surfaces [12]. This limits the diagnostic utility of the blood culture to clinicians required to make decisions concerning the management of the febrile child in real time, which in turn increases the importance of sufficient observation time, blood/urine investigations, and clinical judgement.

With the potential over-treatment of febrile children on the one hand and the prospect of failing to identify potentially life-threatening SBIs on the other, a lack of timely and reliable indicators of febrile aetiology, coupled with a natural tendency for risk aversion when treating children, has resulted in a substantial financial burden to healthcare systems worldwide. However, to date, just a handful of studies, predominantly USA based and conducted between 6 and 25 years ago in young children, have examined the economic impact of paediatric febrile illness [13–16].

Using a bottom-up time-driven and activity-based costing model (TDABC), the aims of this research were to (1) estimate the economic impact of managing febrile illness episodes in children of all ages and presenting complaints, in an NHS paediatric ED setting; (2) identify how management practices and costs vary with factors including patient age, and the experience of treating clinicians; and (3) provide insights regarding where any diagnostic advances currently under development, including molecular diagnostics, protein biomarkers, and point-of-care (POC) testing technologies, are likely to yield the greatest clinical and socioeconomic value, by reducing clinical uncertainty increasing confidence to withhold antibiotics.

## Methods

### Participants and methods

This study applies time-driven activity-based costing (TDABC), a bottom-up approach to healthcare costing, which maps pathways observed during routine clinical practice, identifies all points and durations of interaction therein, and assigns time-dependent costs to each constituent. The costs of non-time-dependent activities, including tariff-based ancillary investigations, are subsequently added to provide a representative activity-weighted cost per completed treatment episode.

A total of 8552 consecutive febrile children, with a temperature above 38 °C at presentation or below 38 °C with an unverified parent-reported history of fever up to 3 days previous, were prospectively identified. All children visited Alder Hey Children’s NHS Foundation Trust, a large paediatric specialist care provider in the North West of England, between 1 September 2012 and 31 August 2013. Children were excluded (1) if data concerning key components of their stay, including the treatments provided, or healthcare personnel seen, were missing or incomplete or (2) if there were pre-existing medical conditions likely to modify ED care pathways from those of the average ‘otherwise well’ patient, including paediatric oncology patients.

**Fig. 1:**
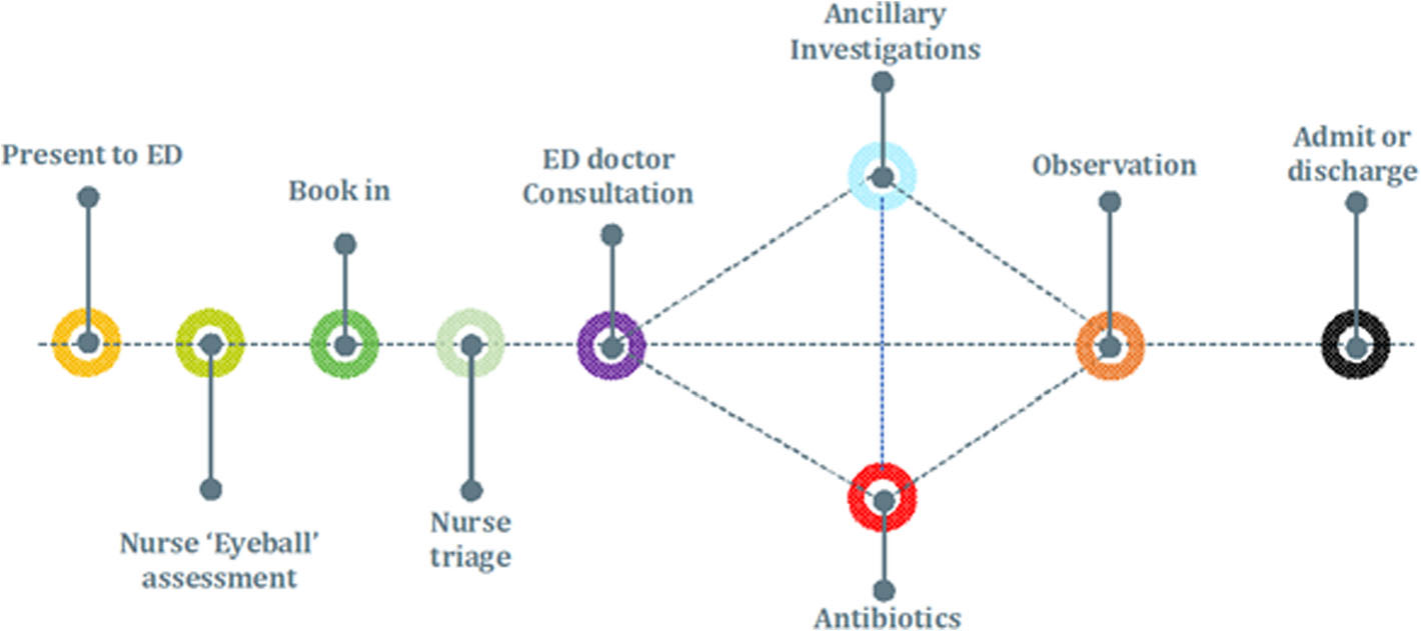
Clinical pathway of paediatric febrile illness used for patient-level costing

A schematic of the clinical pathway used for this study is provided in Fig. 1. Children were initially seen by a qualified ED nurse who conducted an initial evaluation, using the Manchester Triage System (MTS) [17]. MTS assessments follow a flow chart based on the patient’s reason for contacting the ED. The chart begins by identifying possible criteria indicating life-threatening conditions for the patient, and if none of these conditions are present, the nurse continues along the flow chart asking questions until the nurse assigns the patient an appropriate category. The nurse’s experience can contribute to the assessment, but on the other hand, the risk of the nurse missing serious conditions is reduced because the flow chart forces the nurse to ask key questions and make vital inquiries. Children were triaged as green ‘standard’, yellow ‘urgent’, orange ‘very urgent’, or red ‘immediate attention’. For several children, borderline ‘yellow/red’ or ‘orange/red’ categories were applied. This was a result of uncertainty during triage, and such children had their MTS classification amended with increased or reduced urgency following a second opinion with a nurse or clinician. Diagnostic categories, defined as definite bacterial, probable bacterial or bacterial syndrome with low/no inflammatory markers, definite viral, probable viral, or viral syndrome with no/high inflammatory markers, trivial illness, inflammatory illness, and unknown/insufficient information, were applied retrospectively, based on an adapted algorithm from Herberg et al. [18]. In any instance where uncertainty or disagreement occurred regarding the appropriate classification, these cases were marked and decided upon by two consultants specialising in paediatric infectious disease. All cases had notes, including CRP, neutrophils, and sterile site pathogenic bacteria recorded such that diagnosis classifications could be quality checked, to ensure consistency. For this analysis, definite bacterial, probable bacterial and bacterial syndromes with low/no inflammatory markers, were collectively defined as ‘bacterial aetiologies’, while definite viral, probable viral, and viral syndromes with no/high inflammatory markers were collectively defined as ‘viral aetiologies’. Like other studies [19], the prescription of antibiotics for patients with anything other than a bacterial aetiology of fever was, for this study, defined retrospectively as ‘potentially avoidable’.

Because time stamps documenting the duration of contact with healthcare personnel for various treatments and investigations are not routinely collected as part of NHS electronic patient records, these were imputed in one of two ways. Firstly, estimates were provided by staff actively involved in the provision of ED care. Secondly, prospective time-in-motion data were collected for a representative cohort of 71 febrile children presenting to Alder Hey Children’s NHS Foundation Trust ED between January 6 and February 12, 2017. Four 5th-year medical students collected the data by ‘shadowing’ patients reporting to the book-in desk with fever as a symptom. Additionally, any patients suspected of fever by clinical teams (such as the nurse performing an initial visual assessment) were additionally identified. The researchers followed patients through the ED, documenting all points of interaction with healthcare professionals using a stopwatch and a pre-designed case report form. Data were collected in four hourly blocks during the day (8 a.m.–4 p.m.), evening (4 p.m.–12 a.m.), and early morning (12 a.m.–4 a.m.), 7 days a week. All children with a suspected fever were observed from the point of visual assessment, and their experience in the ED, timed using a stopwatch and documented in Microsoft® Excel. For any events which were not observed during implementation of the time-in-motion study, including clerical and administrative tasks such as writing up patient notes, these were estimated following a Delphi panel approach. In all such cases, a number of estimates were obtained and the average time was used because tasks, such as inserting a cannula for example, can be expected to take varying lengths of time depending upon factors such as experience, co-operation of the child, state of hydration, or vascular filling. All timings used are provided in Table 1.

**Table 1:** Staff time associated with components of the paediatric febrile illness pathway

Activity	Mean duration (min)
Triage time (nurse)*	4.5
Clinician consultation time (MTS green)*	16.2
Clinician consultation time (MTS yellow)*	19.4
Clinician consultation time (MTS orange)*	21.1
Clinician consultation time (MTS red)*	22.7
Clinician time—writing up patient notes^#^	10
Order blood/urine culture (clinician)^#^	10
Arrange X-ray (clinician)^#^	6
Book patient into the ED (receptionist)^#^	2
Refer patient to other specialties (clinician)^#^	20
Insert cannula (clinician)*	20
Provide antibiotics/other medicines (nurse)^#^	5
Visual assessment triage (nurse)*	2
Interpret results of ancillary investigations (clinician)^#^	10

*Collected during time-in-motion study

^#^Estimate provided by ED consultants

### Unit costs

Hourly salaries for healthcare personnel were provided by the patient-level costing department at the Trust. Except for clinicians, salaries for those working either (1) at the weekdays between 7 p.m. and 7 a.m. or (2) at the weekend had their hourly rate increased in line with NHS guidance on working unsocial hours [20]. Costs for non-time-driven activities, including laboratory-based investigations, were obtained from the Trust’s finance department and NHS reference costs 2015/16 [21].

Pharmaceuticals were assigned unit costs from the British National Formulary. As data concerning the precise antibiotics provided to patients were not available, we assumed that antibiotic prescribing was in line with the recommendations provided within NICE CG160 [22]. Namely, where intravenous (IV) antibiotics were prescribed, both a third-generation cephalosporin (cefotaxime, ceftriaxone) and an anti-listeria agent were provided (amoxicillin, ampicillin) for infants under 1 month, and a third-generation cephalosporin alone if more than 1 month. In cases of empiric IV antibiotic therapy, it was assumed that a third-generation cephalosporin directed against *Neisseria meningitidis*, *Streptococcus pneumoniae*, *Escherichia coli*, *Staphylococcus aureus*, and *Haemophilus influenzae* type b was provided. Where oral antibiotics were prescribed, it was assumed that amoxicillin or cephalexin was provided as per local antimicrobial guidance.

Costs incurred during inpatient stay were obtained from NHS reference costs 2015/16. The tariff HRG PW20C (paediatric fever of unknown origin, CC score = 0) was utilised to reflect a 3-day short stay inpatient admission. As children could be admitted for anywhere between 1 and 72 h under the reference tariff, this figure was divided through by 72 and multiplied by the number of hours of inpatient admission. Patients who exceeded the 3-day limit incurred an excess bed day charge which was applied from the fourth day until discharge [21]. Finally, indirect costs were estimated for each patient, using the ‘full absorption approach’. This included the anticipated use of facilities, such as toilets, and the time of administrative staff typing up and sending discharge notes to the patient’s general practitioners. Societal costs, including parental absence from work, and children’s absence from school were not included, as the analysis was conducted from a healthcare provider perspective. Due to the short time frame of the analysis, costs were not discounted. All unit costs were in 2017 prices and are provided within Table 2.

**Table 2:** Unit costs by component of paediatric febrile illness pathway

Item	Unit cost
Investigations (per test)	
Amylase	£6.00
Bacterial PCR	£158.00
Bilirubin	£6.00
Biochemistry profile	£8.00
Blood albumin	£6.00
Blood glucose test	£6.00
Blood culture	£35.00
Blood gas^#^	£7.00
Blood taken	£3.00
Calcium profile	£7.00
Clotting screen	£5.00
Creatinine	£6.00
CRP	£6.00
CSF	£6.00
CT scan (head)	£201.00
ECG	£33.00
ENT swab	£19.00
ESR	£4.00
FBC	£3.00
Glandular fever screen	£4.00
Group and save	£12.00
LFTs	£7.00
Magnesium	£6.00
Malarial parasite test	£21.00
Measles PCR	£55.00
Meningo pneumo PCR	£25.00
Meningococci screen	£6.00
Mycoplasma SER	£23.00
Pertussis swab	£9.00
Phosphate	£6.00
Rapid Strep test	£9.00
Renal profile	£46.00
Respiratory PCR	£117.00
RSV screen	£12.00
Ultrasound	£55.00
Urinalysis^#^	£8.00
Urine albumin	£6.00
Urine culture^#^	£8.00
Urine dipstick^#^	£6.00
Urine sample	£8.53
Virus PCR	£56.00
X-ray	£46.00
Antibiotics (per dose/course)	
Amoxicillin 125 mg (suspended)*	£1.16
Amoxicillin 125 mg (IV)*	£4.34
Amoxicillin 250 mg (susp.)*	£1.33
Cefotaxime 195 mg (IV)*	£0.48
Cefotaxime 575 mg (IV)*	£0.66
Nurse time (per hour)	
Band 5	£15.43
Band 6	£18.95
Band 7	£22.50
Band 8a	£27.39
Doctor time (per hour)	
FY1/FY2	£24.24
ST1-3	£30.79
APNP	£27.39
Registrar	£39.02
Consultant	£76.11
Referral to other specialties	
Surgery	£178.55
Medicine	£272.74
ENT	£146.92
Neuro	£411.78
Inpatient admission	
Short stay (HRG PW20C, 3 days non-elective stay)^#^	£1712
Excess bed day charge^#^	£462

Unit costs provided by Alder Hey Finance Team unless otherwise stated

^#^NHS Reference costs 2016

*British National Formulary 2017

### Outcomes and statistical analysis

We present summary statistics to describe the characteristics of participants. Categorical variables were summarised by frequency and percentage, while continuous variables were reported as mean, standard deviation (SD), median, interquartile range (IQR), and minimum and maximum values. Our primary outcome was the ‘cost per completed febrile illness episode’, with an ‘episode’ defined as the period from booking in to the ED to final discharge, enabling the possibility for re-attendances to be included. We additionally performed sub-group analyses to account for patient and healthcare provider heterogeneity. As our primary outcome data were both non-normally distributed, and characterised by sub-groups of unequal size, the Kruskal-Wallis test was applied to assess statistical significance, with Dunn’s post hoc pairwise comparison (adjusted by the Holm FWER method) used to determine where significant differences were present. Results were reported as *p* values and considered statistically significant at the standard 5% level. Multivariate regression analysis using a generalised linear model (GLM) was performed to estimate conditional mean health expenditure and identify covariates associated with increased healthcare utilisation. Because several prior studies have demonstrated that the gamma family with a log error link is not only robust, but also the most commonly applied approach in healthcare cohorts in which positive and skewed healthcare costs are guaranteed [23, 24], our analysis also assumed a gamma error distribution with log link.

Finally, because all timings employed within the TDABC were estimates, and therefore subject to one or more of (1) sampling bias, (2) Hawthorne effects, or (3) reporting bias, a distribution of credible times for each patient interaction with healthcare personnel was used in the time-driven and activity-based costing, to reflect the uncertainty inherent to sampling. For all parameters contained within the time-driven and activity-based costing, continuous variables (time in consultation with clinician, days spent as inpatient) were randomly sampled from gamma distributions as explained by Briggs [25]. Dichotomous variables (percentage of triage assessments performed by band 5/6 nurses) were sampled from representative beta distributions constructed from the sample data available, as explained in previous work by Briggs et al. [26]. For estimates reliant on expert opinion, which were not observed during the time-in-motion study due to a low frequency of occurrence, uniform distributions were sampled in the absence of information concerning the true sample mean and variance. In choosing this distribution, we combined and ranked response data from all healthcare professionals (of varying roles and experience) surveyed, to define lower and upper limits or ‘bounding’ criterion. Once responses were provided, respondents were informed of responses by other respondents to gauge their belief in the credibility of different responses and ensure that the distributions utilised were plausible. GLM regression modelling was subsequently replicated for 100 bootstrapped costing datasets randomly utilising parameter values from all plausible distributions, for all variables, to assess the sensitivity of the primary outcome, the cost per febrile illness episode, and the resulting GLM coefficients, to changes in the values of underlying input parameters. Details of all distributions utilised are provided in Table 3. All analyses were performed using STATA 14 (StataCorp LP, USA) and Microsoft® Excel™ (Redmond, WA).

**Table 3:** Distributions used for probabilistic sensitivity analysis

Parameter	Distribution
Time (hours)	
Nurse triage	Gamma (4.69, 0.01)
Proportion performed by band 6 nurses	Beta (16, 55)
Proportion performed by band 5 nurses	1-Beta (16, 55)
Clinical consultation	Gamma (3.9, 0.04)
Clinician writing up patient notes	Uniform (1, 20)
Arrange blood/urine culture	Uniform (1, 25)
Arranging X-ray	Uniform (1, 30)
Receptionist booking patient in	Uniform (1, 5)
Clinician arranging referral	Uniform (1, 25)
Clinician cannulating child	Uniform (5, 35)
Nurse providing antibiotics to child	Uniform (1, 10)
Visual assessment by nurse	Uniform (0.5,5)
Days spent as inpatient (if admitted)	Gamma (3.72, 1.03)
Salary (cost/hour)	
Nurse (band 5)	Uniform (13.36, 17.5)
Nurse (band 6)	Uniform (16.14, 21.77)
Nurse (band 7)	Uniform (19.34, 25.67)
Nurse (band 8a)	Uniform (24.8, 29.99)
Foundation year doctor	Uniform (22.5,26)
ST1-3	Uniform (27, 30.8)
APNP	Uniform (24.8, 29.99)
Registrar	Uniform (36, 41)
Consultant	Uniform (64.8, 87.4)

## Results

### Descriptive statistics

Eight thousand five hundred fifty-two individual ED attendances were identified over the study period, with 2034 excluded from the analysis due to incomplete data or failing to meet our inclusion criteria. This resulted in a complete dataset of 6518 observations (Table 4). There was no significant difference in observable characteristics between those included and excluded, including but not limited to age, final diagnoses, MTS classification, and temperature.

**Table 4:** Descriptive statistics of the study participants

	Mean (SD)	Median (IQR)	Min	Max
Age	3.28 (3.09)	2.17 (3.5)	4 days	15.98 years
Gender male (freq)	53.5% (3484)	–	–	–
Temperature	38.7 (1.07)	38.6 (1.7)	35	41.4
Respiratory rate (bpm)	29.95 (9.23)	28 (8)	14	188
Pulse (bpm)	138.7 (25.98)	138 (37)	22	250
Manchester Triage Scale (MTS) classification				
MTS green (freq)	47.52% (3097)	–	–	–
MTS yellow (freq)	8.88% (579)	–	–	–
MTS yellow/red (freq)	0.17% (11)			
MTS orange (freq)	17.06% (1112)	–	–	–
MTS orange/red (freq)	23.03% (1501)			
MTS red (freq)	0.39% (27)	–	–	–
MTS not recorded (freq)	2.9% (191)	–	–	–
Timings				
Time between booking and triage (min)	15.3 (14.7)	11 (18)	0	71
< 10	47.8%			
11–20	24.1%			
21–40	20%			
41–60	5.6%			
> 61	2.5%			
Time between triage and consultation (min)	67.9 (52)	55 (65)	0	609
< 30	26.9%			
31–60	27.7%			
61–120	30.8%			
121–180	11.4%			
181–240	2.6%			
> 240	0.6%			
Time in ED post consultation (min)	68.4 (70.6)	45 (72)	0	630
< 30	43.5%			
30–60	15.1%			
61–120	24.8%			
121–180	9.7%			
> 181	7%			
Total time in ED (min)	151.6 (81.3)	135 (98)	16	729
< 60	8.3%			
61–120	32.7%			
121–240	46.9%			
241–360	9.6%			
> 361	2.5%			
Inpatient length of stay (days)				
Not hospitalised	93.51%			
1–3	3.42%			
4–7	2.43%			
8+	0.63%			
Re-attendance (freq)	3.43% (224)	–	–	–
Afterhours (freq)	88.9% (5798)	–	–	–
Winter (freq)	60.1% (3918)	–	–	–
Reviewing clinician				
APNP	2.73% (178)	–	–	–
Consultant	7.99% (521)	–	–	–
Foundation years 1 and 2	0.91% (59)	–	–	–
Registrar	22.05% (1437)	–	–	–
ST1-3	66.32% (4323)			

The mean (median) age of children included was 3.28 (2.17) years, with 53.5% male and 46.5% female. At presentation, 47.52% of children were triaged as green ‘low risk’ cases using the Manchester Triage System (MTS) [17], 8.88% as yellow, 0.17% as yellow/red, 17.06% as orange, 23.03% as orange/red, and 0.39% as red (high risk). MTS classifications were not recorded in 2.9% of patients. Most patients (66.32%) were treated by specialty doctors (ST1-3), followed by registrars or ST4–8 (22.05%), consultants (7.99%), APNPs (2.73%), and Foundation year 1 and 2 doctors (0.91%). The mean (median) time was 15.3 (14.7 min) between booking and triage, 67.9 (52 min) between triage and clinical consultation, and 68.4 (70.6 min) between consultation and discharge. Total mean (median) time in the ED was 151.6 min (81.3 min). Approximately 6.46% of patients were admitted as inpatients, 1.42% of which for a single day, 29.78% 2 days, 21.51% 3 days, and 47.28% > 4 days.

### Determinants of patient-level costs

Table 5 provides details of patient-level resource use and costing. Those aged 0–3 months exhibited a mean treatment cost of £1000.28, [95% CI £82.89–£2993.37], over sixfold higher than the least costly group, aged 3–6 years, (£158.97 [95% CI £20.43–£1596.43]). Use of blood cultures (*p* = 0.0312), urine samples, inpatient admission rates, and inpatient length of stay (*p* = 0.0001) were all statistically significantly increased for those aged 0–3 months, versus all other age groups, as shown in Table 6.

**Table 5:** Health service costs of paediatric febrile illness by sub-group

	Number	Mean	Std. dev	95% CI	Median	IQR	*p* value*
Age							
0–3 months	129	£1000.28	£1469.98	£82.39–£2993.37	£76.65	£1834.10	
3–6 months	281	£522.33	£1737.66	£122.08–£2123.51	£53.63	£55.70	
6–12 months	1041	£205.28	£585.18	£28.26–£734.39	£51.29	£21.50	
1–3 years	2498	£190.44	£594.95	£13.22–£643.89	£51.64	£21.60	
3–6 years	1547	£158.97	£501.82	£20.43–£1596.43	£51.29	£19.80	*p* = 0.0001
6–10 years	707	£165.92	£485.04	£11.14–£843.02	£52.98	£20.70	
10–16 years	315	£408.32	£1030.12	£44.97–£2188.27	£55.55	£40.90	
Gender							
Male	3482	£210.17	£600.23	£38.45–£818.68	£51.29	£21.50	
Female	3036	£238.90	£835.77	£14.13–£924.63	£53.16	£23.10	*p* = 0.0001
NICE NG51 heart rate red flag [27]							
Yes	2797	£259.40	£848.10	£21.76–£1015.89	£54.03	£24.60	*p* = 0.0001
No	3721	£196.59	£604.38	£18.36–£699.74	£50.87	£20.30	
NICE NG51 respiratory rate red flag [27]							
Yes	394	£493.92	£1035.52	£89.16–£2011.32	£66.67	£70.45	
No	6124	£206.15	£691.06	£23.71–£737.44	£51.29	£21.50	*p* = 0.0001
Clinical grade							
APNP	178	£109.52	£312.67	£12.74–£741,65	£48.01	£21.80	
Consultant	521	£315.13	£1344.91	£25.76–£1536.36	£73.23	£40.70	*p* = 0.0001
FY 1 and 2	59	£731.78	£913.38	£97.91–£1125.77	£327.98	£49.90	
Registrar	1437	£255.40	£702.86	£19.40–£1045.91	£54.49	£23.80	
ST1-3	4323	£199.68	£615.00	£12.51–£721.02	£49.77	£28.05	
Afterhours							
Yes	5798	£222.22	£726.36	£14.77–£776.64	£51.92	£22.40	
No	720	£234.19	£664.61	£11.96–£913.33	£51.65	£22.00	*p* = 0.0018
MTS classification							
Green	3098	£121.78	£390.33	£15.81 - £400.93	£49.43	£19.05	
Yellow	579	£424.43	£1027.90	£340.69–£508.17	£63.10	£557.35	
Yellow/red	10	£85.71	£95.24	£71.73–£99.42	£52.33	£16.50	*p* = 0.0001
Orange	1112	£487.16	£1209.15	£416.08–£558.24	£68.86	£77.05	
Orange/red	1502	£152.13	£491.60	£123.44–£170.56	£51.84	£17.20	
Red	26	£549.42	£813.99	£236.47–£862.35	£76.88	£1165.85	
Not recorded	191	£292.01	£966.43	£154.93–£429.09	£50.87	£20.40	
Final diagnosis							
Bacterial infection/syndrome	460	£988.19	£1781.97	£86.89–£2971.08	£77.95	£1757.35	
Viral infection/syndrome	1595	£294.52	£797.43	£18.92–£1082.33	£51.64	£24.25	
Inflammatory infection/syndrome	74	£582.58	£1302.26	£37.60–£1516.05	£63.44	£1140.65	*p* = 0.0001
Other or trivial infection	130	£390.06	£786.27	£22.34–£1243.30	£64.04	£187.15	
Unknown cause	4259	£103.06	£286.52	£12.40–633.87	£51.29	£18.60	

*Kruskal-Wallis test

**Table 6:** Health service utilisation by patient age and MTS score

	Inpatient	Length of stay (days)^#^	Any test	Blood culture	X-ray	Urine sample	Review by consultant
Age							
0–3 months	34.11%	5.67	51.16%	28.70%	9.30%	39.53%	10.07%
3–6 months	15.66%	5.34	40.92%	11.03%	12.10%	32.74%	5.69%
6–12 months	6.34%	3.83	31.98%	2.01%	9.12%	23.24%	8.64%
1–3 years	5.36%	4.05	29.74%	2.52%	10.88%	18.37%	7.64%
3–6 years	4.01%	4.02	28.70%	3.03%	9.43%	13.70%	8.14%
6–10 years	4.53%	3.78	34.08%	3.67%	9.61%	17.25%	8.76%
10–16 years	7.96%	4.73	42.22%	8.88%	10.15%	15.87%	7.3%
*p* value	0.0001^§^	0.0001*	0.0001^§^	0.0001^§^	0.5370^§^	0.0001^§^	0.1342^§^
MTS classification							
Green	2.61%	3.88	24.59%	1.51%	5.68%	16.17%	8.06%
Yellow	13.64%	4.64	43.52%	7.42%	11.91%	23.48%	9.32%
Orange	17.27%	4.23	44.6%	10.07%	23.2%	19.15%	8.45%
Red	30.77%	2.63	26.92%	15.38%	11.53%	11.53%	23.07%
*p* value	0.0001^§^	0.0001^#^	0.0001^§^	0.0001^§^	0.0001^§^	0.0023^§^	

^#^Mean length of stay among those admitted for at least 1 day

*Kruskal-Wallis test

^§^Chi-squared test

The distribution of MTS classifications was approximately equal across all age groups, except for those aged 0–3 months, 74.41% of which were triaged as yellow or higher. As expected, overall healthcare expenditure increased with increasing MTS severity, from £121.78 per patient (green), £424.43 (yellow), £487.16 (orange), and £549.42 (red), the majority of which as a direct result of increasing rates of inpatient admission. A one-step increase in triage category, from green to yellow, resulted in a 422% increase in inpatient admission, a 19.6% increase in length of stay for those admitted, and a 391% increase in use of blood cultures. In terms of final diagnoses, bacterial infections were most commonly observed in those aged 0–3 months (15.5%), 3–6 months (11.03%), and 10–16 years (11.74%); however, the only significant difference was when comparing these groups to those aged 1–3 years (4.6%), *p* < 0.05. Those with bacterial aetiologies of fever exhibited over threefold higher management costs than those with viral aetiologies (£988.19 vs. £294.52).

### Antibiotic prescribing patterns

Approximately 32.4% of febrile children were prescribed antibiotics, of whom 7.05% were retrospectively diagnosed with bacterial aetiologies of fever. Approximately 14.9% of patients were retrospectively classified as having inflammatory, 10.8% as trivial, and 6.6% as viral aetiologies of fever (probable, definite and viral syndromes) were prescribed potentially avoidable antibiotics, if a means of distinguishing these from bacterial causes of infection has been available (Table 7). Analysing children with viral causes of fever who were triaged as MTS green or yellow (those not deemed to require very urgent or immediate care), those receiving antibiotics spent an additional 53.9 h as inpatients (57.1 vs. 3.2 h) compared to children with viral aetiologies of fever, triaged MTS green or yellow, who were not prescribed antibiotics. This resulted in a 9.9-fold increase in management costs for those who received potentially avoidable antibiotics (£1392.30 vs. £140.10) as shown in Table 8, the majority of which attributable to the costs of inpatient or short stay beds for observation.

**Table 7:** Antibiotic prescribing rates differentiated by age and final diagnosis

	Receiving antibiotics								
	Total (%)	0–3 months (%)	3–6 months (%)	6–12 months (%)	1–3 years (%)	3–6 years (%)	6–10 years (%)	10–16 years (%)	*p* value^#^
All	32.4	27.9	24.2	24	31.9	37	34.5	40.3	0.0001
Bacterial	89.6	85	96.8	84.3	93	89	87.7	91.9	0.3610
Viral	6.6	20.8	10	3.2	9.4	4	2.60	5.7	0.0001
Inflammatory	14.9	0	0	0	9.5	17.2	23.1	12.5	0.9330
Trivial	10.8	0	50	0	9.7	8.1	20	5.3	0.0820
Unknown	36.4	17.3	19.2	25.5	35.7	43.3	42.2	48.1	0.0001

^#^Chi-squared test

**Table 8:** Treatment costs differentiated by age, final diagnosis, and antibiotic status

	Viral		Trivial		Inflammatory		Bacterial	
Antibiotics given?	Yes	No	Yes	No	Yes	No	Yes	No
All*	£1392.30	£140.10	£324.49	£224.54	£185.08	£669.86	£755.03	£747.43
0–3 months	£2842.60	£479.65	N/A	£113.81	N/A	£50.87	£2476.96	£2419.07
3–6 months	£1969.38	£142.81	£50.39	£334.50	N/A	£65.92	£1078.39	£60.78
6–12 months	£2452.83	£159.57	N/A	£58.63	N/A	N/A	£376.20	£774.53
1–3 years	£687.02	£151.09	£2223.43	£256.88	£51.43	£390.81	£883.52	£278.09
3–6 years	£1201.76	£123.97	£58.69	£196.88	£54.52	£355.06	£450.45	£586.77
6–10 years	£1575.80	£63.65	£51.46	£87.65	£475.93	£447.47	£416.84	£672.95
10–16 years	£2603.54	£143.37	N/A	£401.88	£101.95	£4842.32	£1484.10	£694.91

*MTS green and yellow only

### Determinants of increased healthcare expenditure during paediatric febrile episodes

Based on generalised linear modelling, compared to the reference group of those aged 1–3 years, those aged 0–3 months experienced a 3.54-fold [95% CI 2.59–4.85-fold, *p* < 0.0001] increase in healthcare resource use. The presence of a NICE NG51 respiratory rate red flag [27] increased costs by 72.1% (*p* < 0.0001) (Table 6). Other factors associated with increased resource use included treatment by Foundation year 1/Foundation year 2 (FY1/FY2) doctors, which were increased 3.19-fold, relative to the consultant reference group, *p* < 0.0001. When considering only non-urgent children, triaged as green using the MTS, FY1/FY2 doctors exhibited a 7.98-fold increase in costs of management, relative to consultants (*p* < 0.0001). FY1/FY2 doctors recorded the highest rates of inpatient admission, ancillary investigations, and referring children to other specialties. Comparing resource use for FY1/FY2 doctors working out of hours and those working during regular hours, where the availability of ancillary investigations may be reduced, there was no significant difference (*p* = 0.9626). Factors including male gender and being treated by an APNP were shown to reduce costs by 15.1% (*p* = 0.0241) and 42.7% (*p* = 0.0112) respectively, as shown in Table 9.

**Table 9:** Determinants of healthcare resource use for paediatric febrile episodes

Coefficient	Ln(β)	Exp (β)	95% CI (β) low	95% CI (β) high	*p* value
0–3 months	1.265	3.543	2.589	4.85	0.001
3–6 months	0.791	2.207	1.544	3.155	0.001
6–12 months	0.171	1.186	0.924	1.524	0.180
3–6 years	− 0.164	0.848	0.705	1.021	0.082
6–10 years	− 0.046	0.954	0.738	1.235	0.724
10–16 years	0.656	1.927	1.399	2.654	0.001
Gender (male)	− 0.163	0.849	0.736	0.978	0.024
Time from book-in to triage	− 0.005	0.994	0.990	0.999	0.013
NICE HR	0.034	1.034	0.894	1.197	0.644
NICE RR	0.543	1.721	1.289	2.299	0.001
Time from triage to call in	− 0.001	0.999	0.997	1.000	0.357
APNP	− 0.555	0.573	0.374	0.878	0.011
FY1/FY2	1.161	3.193	2.017	5.055	0.001
ST1-3	− 0.161	0.851	0.670	1.081	0.187
Registrar	− 0.068	0.933	0.719	1.212	0.608
After hours**	0.147	1.159	0.867	1.548	0.317
Winter	− 0.215	0.806	0.695	0.934	0.004
MTS yellow	0.868	2.382	1.905	2.979	0.001
MTS orange	1.049	2.857	2.397	3.405	0.001
MTS red	1.096	2.992	1.762	5.081	0.001

^*#*^Figures are exponentiated GLM (gamma, log) coefficients, interpreted as *x*-fold increases versus the reference group

*Reference group age = 1–3 years, reference group clinical grade = consultants, reference group MTS classification = green

**Between the hours of 6.30 p.m. and 8 a.m. Monday to Friday, and all-day Saturday, Sunday, and bank holidays

**Fig. 2:**
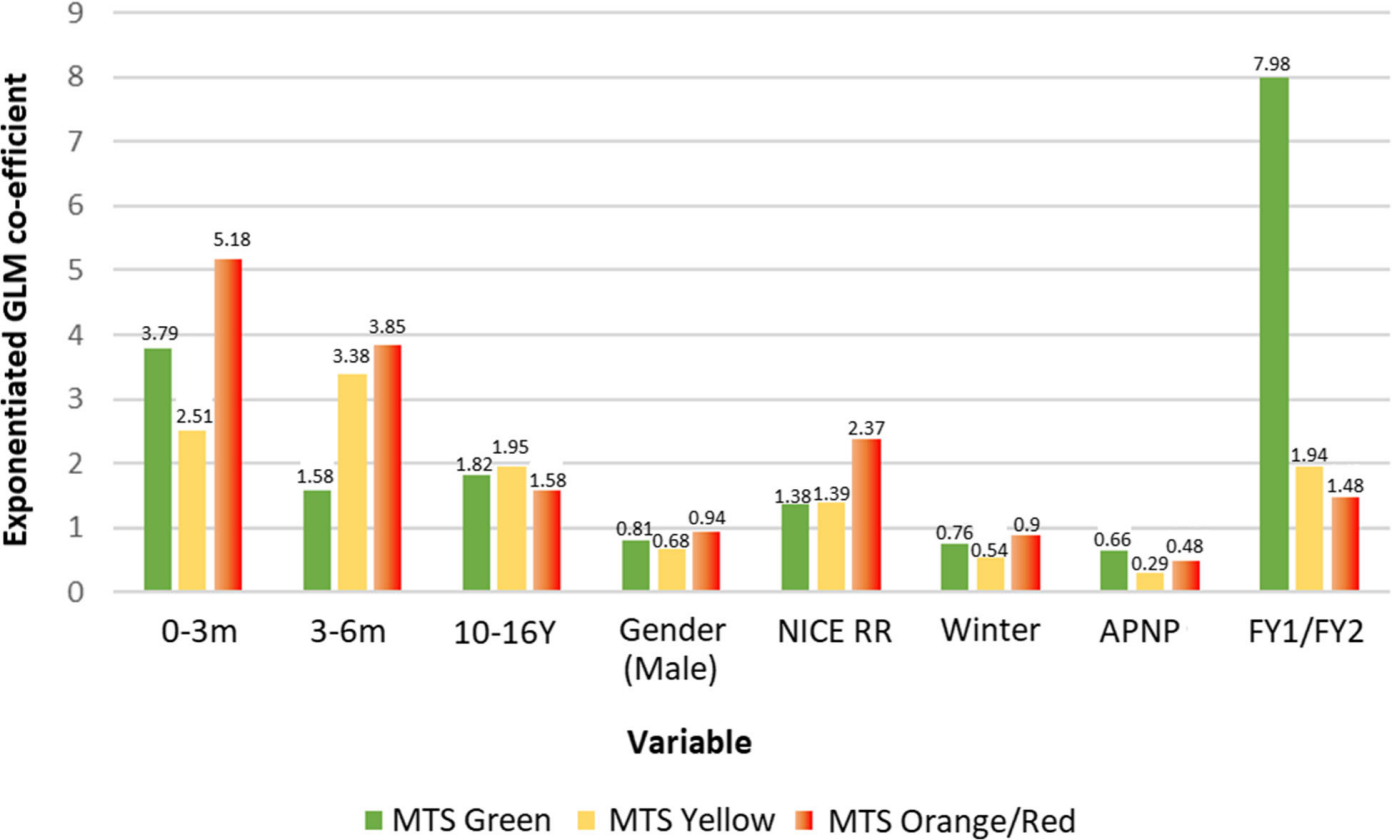
Determinants of healthcare resource use among febrile children of differing clinical risk/urgency

Increasing clinical severity, as proxied by increasing MTS classifications, resulted in significant cost increases of 138.2% (2.38-fold), 185.7% (2.85 fold), and 199.2% (2.99-fold) respectively compared to children triaged as green (all *p* < 0.01). As such, we performed independent GLM regressions for three MTS groups (green, yellow, and orange/red), to account for the possibility that severity of illness may have an important role in determining overall resource use. Similar to the results when pooling children of all severities, those demonstrated in Fig. 2 highlight the consistent importance of ages (< 6 months, 10–16 years), prompting a NICE respiratory rate red flag [27], and being treated by an FY1 or FY2 doctor, suggesting that these are key drivers of increased resource use when managing paediatric febrile illness after taking clinical severity into account.

### Sensitivity analysis

Our findings were insensitive to changes in the values of our input parameters. Following the Monte Carlo simulation and re-running our generalised linear models on 100 bootstrapped datasets, the coefficients listed in Table 10 were obtained. Children triaged as MTS yellow or above, those prompting a NICE NG51 respiratory rate red flag, and those treated by an FY1/FY2 doctor, and treatment of children aged 0–3 months, 3–6 months, or 10–16 years respectively, were statistically significant predictors of increased healthcare costs in 100% of simulations. Conversely, the cost savings associated with male gender and treatment by an APNP remained significant in just 8% and 28.3% of simulations respectively.

**Table 10:** Sensitivity analyses of determinants of healthcare costs for paediatric febrile episodes

	*β* (base-case)^#^	*β* (bootstrapped)	Minimum *β* (% lower)^#^	Maximum *β* (% higher)^#^	Statistically significant* (%)
0–3 months	3.543	3.11	2.16 (39.02%)	3.92 (10.69%)	100
0–6 months	2.207	2.08	1.45 (34.39%)	2.68 (21.55%)	100
6–12 months	1.186	1.27	1.00 (15.75%)	1.54 (29.84%)	38.38
3–6 years	0.848	0.88	0.68 (19.3%)	0.98 (15.77%)	19.19
6–10 years	0.954	1.00	0.74 (22.39%)	1.18 (23.63%)	0
10–16 years	1.927	1.81	1.25 (35.27%)	2.10 (8.98%)	100
Gender (male)	0.849	0.90	0.78 (7.91%)	0.99 (16.64%)	8.08
Time (book-in to triage)	0.994	1.00	0.99 (0.24%)	1.00 (0.65%)	16.16
NICE HR	1.034	1.03	0.89 (14.04%)	1.12 (8.75%)	0
NICE RR	1.721	1.65	1.19 (30.71%)	1.99 (15.60%)	100
Time (triage to call in)	0.999	1.00	1.00 (0.14%)	1.00 (0.13%)	3.03
APNP	0.573	0.69	0.37 (36.23%)	0.99 (72.91%)	28.28
FY1/FY2	3.193	3.29	1.98 (37.94%)	4.06 (27.11%)	100
ST1-3	0.851	0.90	0.72 (15.88%)	1.01 (18.17%)	0
REG	0.933	1.00	0.76 (19.02%)	1.12 (20.10%)	0
After hours	1.159	1.19	0.90 (21.98%)	1.47 (26.54%)	2.02
Winter	0.806	0.79	0.68 (15.08%)	0.89 (10.11%)	98.99
MTS yellow	2.382	2.27	1.77 (25.67%)	2.61 (9.59%)	100
MTS orange	2.857	2.89	2.23 (22.08%)	3.21 (12.43%)	100
MTS red	2.992	4.52	1.95 (34.80%)	6.87 (129.76%)	100
Constant	164.8	143.50	90.33 (45.19%)	179.37 (8.84%)	100

Reference group age = 1–3 years, reference group clinical grade = consultants, reference group MTS classification = green

^#^Figures are exponentiated GLM (gamma, log) coefficients, interpreted as *x*-fold increases versus the reference group

*Proportion of 100 bootstrapped GLM regressions in which *p* value was < 0.05

## Discussion

This study reports the largest comprehensive, prospective observational study to date, assessing the economic implications of diagnostic uncertainty when managing paediatric febrile illness, in those aged 0–16 years, in an ED setting. In a full cohort analysis on the management of this highly common condition, we demonstrate that the healthcare resources required to manage this condition are both significant and subject to extensive variation, some of which can be explained by the presence of certain patient and healthcare provider characteristics. Infants aged 0–6 months (particularly those aged 0–3 months), those triaged as MTS yellow or above, and those managed by lesser experienced clinicians (FY1 and FY2) required significantly greater resources in the ED. This was primarily a result of increases in observation time for patients and inpatient length of stay, the latter particularly prominent in those receiving antibiotics. In cases of MTS green and yellow viral infections, where antibiotics were potentially avoidable, provided more sensitive and prompt diagnostics had been available at this time, costs increased 9.9-fold (95% CI 6.48–13.2-fold). This was equivalent to an additional £1352.20 spend per patient (all patients pooled), rising to £2363 for infants aged less than 3 months.

Our study had several strengths. We included more than 6500 febrile children over all seasons during a 1-year period, and by applying TDABC methodology, we could achieve significant detail regarding actual resource use. This resulted in an inclusive and representative estimate of the economic impact of paediatric febrile illness to NHS EDs. Capturing model input data using a prospective time-in-motion approach provided confidence regarding the time requirements of essential components of care in the patient pathway. Data regarding these patient touchpoints are not currently available in published literature, and we believe this analysis has filled a gap which may subsequently be used for similar health economic analyses in the future.

Limitations of our study include the fact that presumed viral and bacterial aetiologies of fever were applied retrospectively; therefore, we lacked the benefit of clinical acumen and parental anxiety which could heavily influence the decision to prescribe antibiotics. While we made every effort to minimise bias when coding final diagnoses using the algorithm provided by Herberg et al. [18], there is a possibility that errors could have occurred, which may have affected conclusions regarding potentially avoidable antibiotics in the event of an incorrect diagnosis. However, following random sampling and checking of diagnoses, we believe the likelihood of this to be minimal given the level of detail provided and simplicity in using the diagnosis algorithm. Another potential limitation is the completeness of the dataset, with just under 24% of observations removed due to missing or incomplete data. While it was assumed that these data were missing at random, we cannot be sure of this, and as such, we are unsure how the results may have differed if data for these 2034 children were available. While we made every effort to ensure a thorough approach to capturing NHS resource use, there were also instances where we likely underestimated costs. Our time-in-motion data did not capture information regarding additional consultations and advice from senior members of staff, which are likely to increase the costs of lesser experienced clinicians managing febrile children, nor did it include the societal costs of febrile illness borne by parents, including time off work, especially in the case of hospitalisation. Considering that new diagnostics may result in a reduction in antibiotic use, it is plausible that re-attendances or time observing patients in the department could increase, thereby potentially reducing the value to parents of improved diagnostics. The final limitation of our study concerns the generalizability of the findings to other settings, whether in the UK, Europe, or further afield. Our data were collected from a single site, and our analysis based on local prescribing protocols, as such, the economic value of improving the management of febrile illness in other settings, including the USA, where are a more consultant-led approach may be more common, may differ from those demonstrated here.

Two previous studies have reported healthcare costs for managing children with SBIs, namely UTI [13] and meningitis [14]. Two studies reporting costs of management for children with fever of any cause [15, 16] have been performed in the USA, with data collected at least 5 years ago, in children aged < 3 years and < 90 days respectively, thereby limiting their generalisability. Additionally, one study conducted in Switzerland demonstrates the cost of illness associated with paediatric community-acquired pneumonia in 2010 [28]. However, no study prior to ours has assessed the resource implications of managing fever in a broad and representative cohort of all ages, diagnoses, and types of resource use in Europe.

The finding that infants (particularly those aged < 3 months) tended to require significantly greater ED resources may be explained by increased cautiousness and a lack of symptomatic information directly from the children themselves, when managing febrile infants. Despite most causes of fever in children being self-limiting, the fear of missing life-threatening infection in children with fever remains a persistent problem for clinicians, who have a natural tendency to be risk averse [29]. Commonly reported concerns among clinicians treating febrile children include suspected central nervous system damage (24%), seizures (19%), and death (5%) [30], manifesting in overly aggressive, and often, in hindsight, unnecessary treatment [31]. Additionally, the prevalence of invasive bacterial infections, bacteraemia and bacterial meningitis, is highest in the first 3 months of life, driving clinician behaviour towards a cautious approach in this high-risk group. Clinical prediction rules, such as the Yale observation scale may be useful in these groups, particularly among those with less experience in ruling in/out serious bacterial infections; however, reliability in higher [32] vs. lower income countries [33] is variable, suggesting that these alone may not be enough to fill the diagnostic gap faced by the clinician managing paediatric febrile illness [34].

Though potentially avoidable antibiotic prescribing was lower in our cohort (6.6% viral, 10.8% trivial illness) than in similar studies based in the USA (36%) [35] and Oxford, England (34%) [36], we found that antibiotic prescribing for those with viral causes of fever was highest in those aged 0–3 (20.8%), and 3–6 months (10%) supporting our finding of an increased tendency to be cautious when treating young febrile infants. This resulted in not only a substantial increase in ED resource use, but also likely increased inconvenience and distress to the children and parents involved, due to potentially unnecessary investigations and treatment. Furthermore, excess use of antibiotics is known to contribute to increasing rates of antimicrobial resistance (AMR) [37], an important component of both the clinical and economic impact of AB prescribing which we were unable to quantify in this analysis.

Given the paucity of published evidence, additional research examining the patient-centred and societal implications of current diagnosis and treatment practices when managing the febrile child would add considerable value for those looking to determine the true value of improved diagnostics, which may be capable of better targeting of scarce ED resources. Given the variable performance and accuracy of the MTS triage system in paediatric populations, we believe our finding that costs increased with MTS severity is noteworthy. Recent large-scale validation studies have highlighted the low reliability of the MTS in both younger [17] and older children presenting to the ED with fever [38], with an estimated 54% of children over-triaged when using the MTS [34]. In adult studies, over-triaging by just a single category, from green to yellow, has been shown to increase the use of electrocardiogram (ECG) and laboratory investigations by 261% and 148% respectively [39]. Similarly, in our study, children triaged as yellow experienced a 422% increase in inpatient stay, a 76.9% increase in ancillary investigations, and a 15.6% increase in review by consultants, versus those triaged as green. As the MTS categories yellow, orange, and red represent urgent, very urgent, and immediate attention respectively, these are the groups with the highest probability of SBIs, we believe these are the groups where novel diagnostics should be targeted.

While we found evidence of an increase in healthcare utilisation among the least experienced clinicians (FY1/FY2), just 0.9% of clinicians included in our study were FY1 and FY2 doctors. The results observed in this sample were therefore highly susceptible to bias through a lack of inter-clinician variability, and with a larger sample size may regress towards a lower mean. Additionally, although GLM analyses highlighted a 44.2% increase in time spent in the ED for those treated by FY1 and FY2 doctors when compared to consultants, this was likely due to the need to seek second opinions from more experienced colleagues, something which we were unable to attach costs to. This may also have been because lower acuity patients wait the longest and are more likely to be seen by lesser experienced doctors, as the sickest are re-directed to senior doctors. Because it is likely that any advances in diagnostics are likely to be heavily used by lesser experienced doctors, this could reduce times in the ED, but potentially still increase management costs. This is particularly true if the price of novel POC tests is high, as with multiplex PCR, which may cost the same as a day in the hospital when first released. The price of such tests can however be expected to decrease over time, resulting in savings over the longer term.

## Conclusions

In conclusion, based on a comprehensive and representative sample of febrile children of varying age, presenting complaints, final diagnoses, and treating clinicians, this study has shown that the management of paediatric febrile illness in the ED poses a substantial financial burden. This is predominantly due to the impact of diagnostic uncertainty, which most often leads to in increased observation time and inpatient admission. Children aged 0–6 months, those triaged as MTS yellow and above, and those managed by newly qualified doctors are the most likely to receive additional resources in the ED. After accounting for the severity of illness, precautionary antibiotic prescribing, particularly in younger low acuity children with viral illnesses, is associated with substantial increases in health service utilisation, predominantly because of increases in inpatient admissions. So far, information on potential shifts in infection epidemiology, such as an increase in healthcare-associated infections or reductions in vaccine-preventable infections or increases in invasive disease due to serotype replacement, are unlikely to affect our conclusions. Comparable settings in the UK and elsewhere will likely show similar patterns in resource use. Any advances in diagnostic capabilities, including molecular diagnostics, protein biomarkers, and POC tests would likely yield the potentially greatest efficiency gains in these groups of children, as among these the perceived risks of untimely diagnosis are greatest.

## Abbreviations

95% CI: 95% confidence interval
AB: Antibiotic
AOM: Acute otitis media
APNP: Advanced paediatric nurse practitioner
CRP: C-reactive protein
ECG: Electrocardiogram
ED: Emergency department
FY1/FY2: Foundation year 1/Foundation year 2
GLM: Generalised linear model
IQR: Interquartile range
MTS: Manchester Triage System
NHS: National Health Service
NICE: National Institute for Health and Care Excellence
POC: Point of care
SBI: Serious bacterial infection
SD: Standard deviation
ST1-3: Specialised training years 1–3
TDABC: Time-driven and activity-based costing
USA: United States of America
UTI: Urinary tract infection
